# Bridging multi-hole self-expandable metal stent deployment for hepatic hilar obstruction under endoscopic ultrasound guidance

**DOI:** 10.1055/a-2814-5900

**Published:** 2026-03-09

**Authors:** Junichi Nakamura, Takeshi Ogura, Kimi Bessho, Nga Nguyen Trong, Hiroki Nishikawa

**Affiliations:** 1 13010 Pancreatobiliary Advanced Medical Center, Osaka Medical and Pharmaceutical University Hospital, Osaka, Japan; 2 2nd Department of Internal Medicine, Osaka Medical and Pharmaceutical University, Osaka, Japan; 3 Endoscopy Center, Osaka Medical and Pharmaceutical University Hospital, Osaka, Japan; 4 591366 Gastroenterology, Trong Nam Cancer Hospital, Hanoi, Vietnam

Endoscopic ultrasound (EUS)-guided biliary drainage for hepatic hilar obstruction can be performed using EUS-guided hepaticogastrostomy (EUS-HGS) combined with a bridging technique 1 . Several factors affect the choice of stents for the bridging technique. First, uncovered self-expandable metal stents (UCSEMSs) facilitate the prevention of bile duct branch obstruction. Second, the insertion of the stent delivery system from the left hepatic bile duct to the right hepatic bile duct requires favorable push ability and a fine gauge diameter of the stent delivery system. However, tumor ingrowth limits the utility of UCSEMSs. On the other hand, although fully covered SEMSs (FCSEMSs) can prevent tumor ingrowth, their use is complicated by subsequent bile duct branch obstruction. In addition, since the stent delivery systems of FCSEMSs usually have a large diameter, they might not be suitable as stents in the bridging technique. To overcome these issues, an FCSEMS with side holes (HANAROSTENT Biliary Multi-hole Benefit; M.I. Tech Co., Ltd, Pyeongtaek, South Korea) has been developed (multi-hole self-expandable metal stents [MHSEMS]; Fig. 1 ). The stent is designed to prevent stent migration through small tissue ingrowths that form in the multiple, small side holes along the covering membrane. Additionally, since the stent delivery system is only 5.9 Fr in diameter, it might be suitable for the bridging technique. We present the technical tips for EUS-HGS combined with the bridging technique using the MHSEMS.

**Fig. 1:**
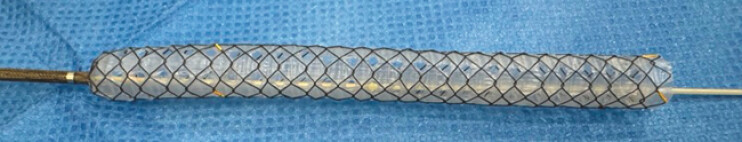
A fully covered self-expandable metal stent with side holes (HANAROSTENT Biliary Multi-hole Benefit; M.I. Tech Co., Ltd, Pyeongtaek, South Korea).

An 81-year-old woman was admitted to our hospital for the treatment of obstructive jaundice due to cholangiocarcinoma. She had previously undergone distal gastrectomy with Roux-en Y reconstruction for distal gastric cancer. Because enteroscopic-guided endoscopic retrograde cholangiopancreatography (ERCP) was unsuccessful, EUS-HGS was performed. After successful bile duct puncture and contrast medium injection, a 0.025-inch guidewire was inserted. Then, the ERCP catheter was inserted and the contrast medium was injected again. Under cholangiography, the hepatic hilar obstruction was observed, and the guidewire was successfully advanced into the right hepatic biliary system ( Fig. 2 ). Next, an uneven ERCP catheter was inserted, and an additional guidewire was deployed ( Fig. 3 ). Subsequently, the MHSEMS insertion into the right hepatic bile duct was attempted. The fine-gauge stent delivery system and its favorable push ability facilitated the successful performance of this procedure ( Fig. 4 ). EUS-HGS using a partially covered SEMS was performed after successful MHSEMS deployment ( Fig. 5 ; Video 1 ). After biliary drainage, obstructive jaundice was successfully treated. During 4-month clinical follow-up until patient death, recurrent biliary obstruction including stent obstruction and migration was not observed.

**Fig. 2:**
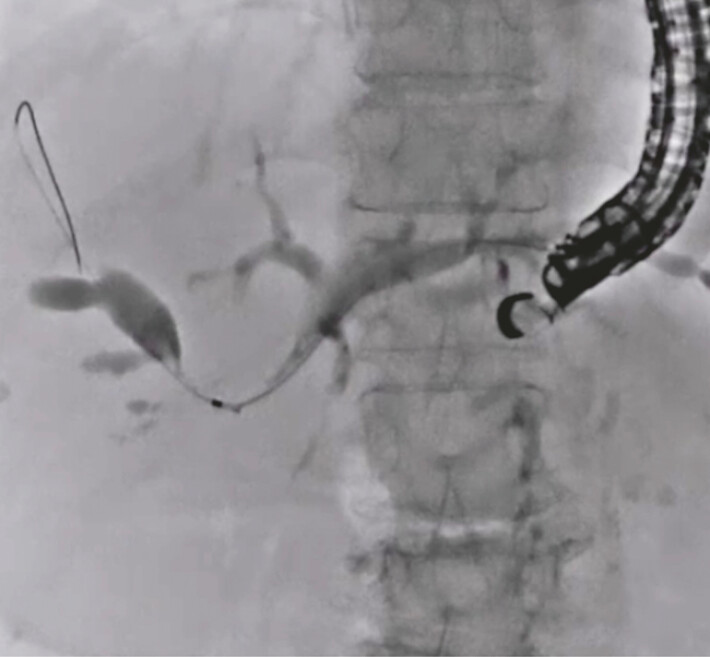
Observation of hepatic hilar obstruction.

**Fig. 3:**
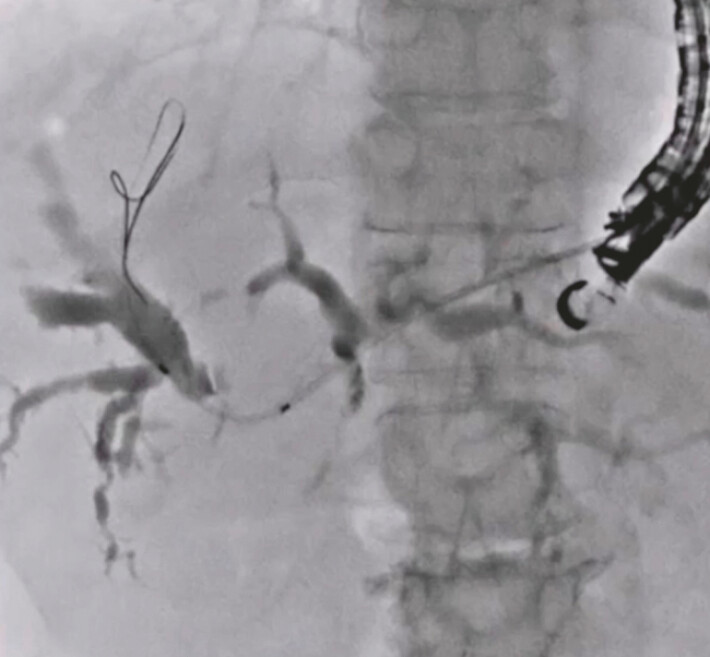
Performance of the double guidewire technique.

**Fig. 4:**
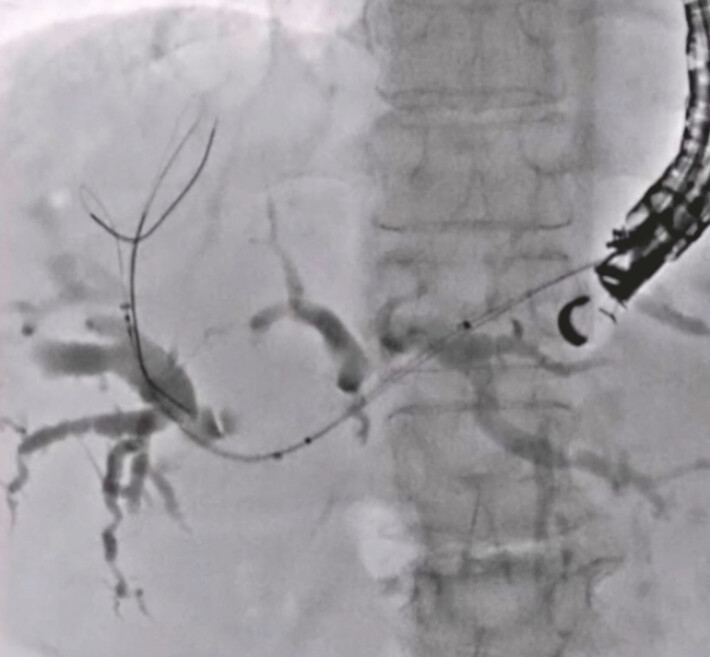
The 5.9-Fr stent delivery system of the fully covered self-expandable metal stent with side holes was inserted into the right hepatic bile duct without stricture dilation.

**Fig. 5:**
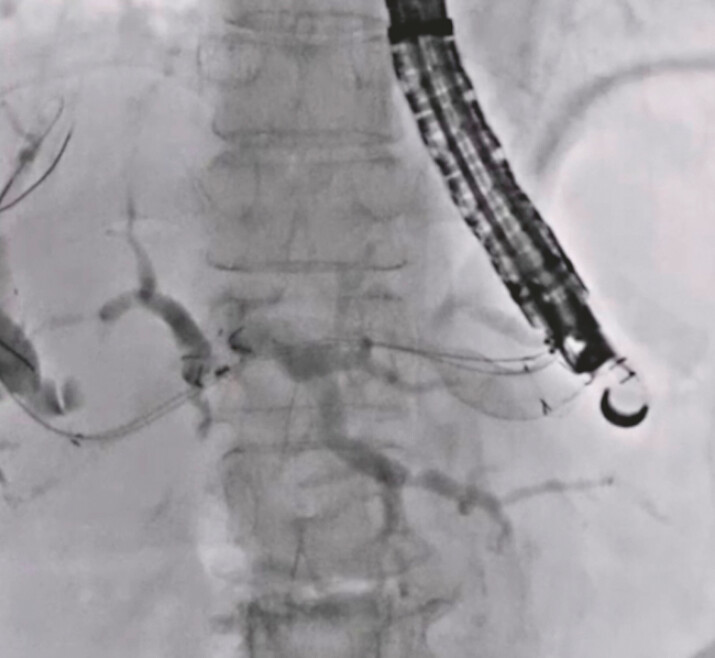
A partially covered self-expandable metal stent was deployed from the intrahepatic bile duct to the stomach.

A multi-hole self-expandable metal stent delivery system was successfully inserted from the right hepatic bile duct to the left hepatic bile duct. Bridging multi-hole self-expandable metal stent deployment for hepatic hilar obstruction under endoscopic ultrasound guidance is performed.Video 1

In conclusion, EUS-HGS combined with a bridging technique using the MHSEMS might be useful, although the validation of this device in a clinical trial is needed.

Endoscopy_UCTN_Code_TTT_1AS_2AH Endoscopy_UCTN_Code_TTT_1AS_2AK

## References

[1] Ogura T Sano T Onda S Endoscopic ultrasound-guided biliary drainage for right hepatic bile duct obstruction: novel technical tips Endoscopy 2015 47 72 75 10.1055/s-0034-1378111 25264761

